# Pediatric high risk neuroblastoma with autologous stem cell transplant – 20 years of experience

**DOI:** 10.1016/j.ijpam.2021.02.006

**Published:** 2021-03-03

**Authors:** Saadiya Khan, Khulood AlSayyad, Khawar Siddiqui, Awatif AlAnazi, Amal AlSeraihy, Ali AlAhmari, Hassan ElSolh, Ibrahim Ghemlas, Hawazen AlSaedi, Abdullah AlJefri, Afshan Ali, Ibrahim AlFawaz, Amani AlKofide, Mouhab Ayas

**Affiliations:** Department of Pediatric Hematology / Oncology, MBC 53, King Faisal Specialist Hospital and Research Center, Riyadh, Saudi Arabia

**Keywords:** High-risk neuroblastoma, Autologous stem cell transplant, Oncology, Pediatric

## Abstract

**Background and Objective:**

Neuroblastoma is the most common extracranial solid tumor found in pediatric patients. High-risk neuroblastoma (HR-NBL) can be characterized by metastasis, age, and other tumor characteristics that result in an adverse outlook for this patient cohort. The standard of care includes induction chemotherapy, surgery, followed by stem cell autologous transplant (ASCT), and later, antidisialoganglioside (anti-GD2) antibodies. In this study, we provide the survival and toxicity data of our HR-NBL patients treated with a single ASCT.

**Methods:**

We retrospectively analyzed pediatric HR-NBL patients treated with single ASCT after a carboplatin, etoposide, and melphalan (CEM) regimen in our institution between January 1993 and December 2014.

**Results:**

There were 99 evaluable patients with male predominance. The median age at diagnosis was 3 years. Most of our HR-NBL patients were stage 4 (88%). All patients received ASCT. Peripheral blood was the graft source in 58% of the patients. Time for hematological count recovery with bone marrow as a graft source was prolonged but not statistically significant when compared with PBSCs. Of all the patients, 58% received radiation therapy to residual disease. Overt secondary leukemia was not seen in any of these patients. Three-year overall survival (OS) was 68.5% ± 5.2% and the 3-year event-free survival (EFS) was (48.3% ± 5.2%).

**Conclusion:**

Our HR-NBL patients tolerated high-dose chemotherapy well followed by single autologous stem cell transplant. Tandem transplant is a feasible option in our patient cohort. Apart from secondary solid tumors, there were no long-term complications seen.

## Abbreviations

NBL: Neuroblastoma
HR-NBL: High-Risk Neuroblastoma
ASCT: Autologous Stem Cell Transplant
EFS: Event Free Survival
OS: Overall Survival
XRT: Radiation Therapy
CR: Complete Response
TBI: Total Body Irradiation

## 1 Introduction

Neuroblastoma (NBL) is a malignancy of the sympathetic nervous system that arises in cells derived from the neural crest. NBL predominantly affects young children.

It is the most common pediatric extracranial solid tumor accountable for about 15% of cancer-related deaths annually [1]. Survival outcomes of NBL have improved over the years and are still in the evolution stage, given the clinical and biological heterogeneity of this disease entity. Most children who present with low and intermediate risk disease have near excellent results. While those who present with high-risk neuroblastoma (HR-NBL) need an intense multimodality approach for cure. For these HR-NBL patients, cure rates are estimated at ≤ 50% [2,3].

The International Neuroblastoma Risk Group stage along with clinical and biological prognostic markers such as age, tumor differentiation, N-MYC status, and ploidy are used for risk assignment. Children who are ≥18 months of age with metastatic disease or those with N-MYC amplification irrespective of age and disease stage (locoregional or metastatic) are classified as HR-NBL [4]. There has been significant improvement in the treatment of these HR-NBL patients with respect to the consolidation phase that includes myeloablative chemotherapy followed by autologous stem cell transplant (ASCT) as well as the postconsolidation phase, which includes immunotherapy [[5], [6], [7]].

Acute toxicities of ASCT can be critical and depend on the types and doses of myeloablative chemotherapy that was used [8,9]. In this study, we aimed to review the outcomes of a single ASCT consolidation therapy as well as a pattern of disease seen with a 3-year follow-up of our patients.

## 2 Methods

Pediatric patients under the age of 14 years with a diagnosis of NBL who underwent ASCT were identified from the clinical database at our institution between January 1993 and December 2014. Medical records were reviewed and data pertaining to patient demographics, disease stage, treatment modalities, treatment-related toxicity [10], and outcomes were collected on a case report form after patient de-identification.

**Fig. 1:**
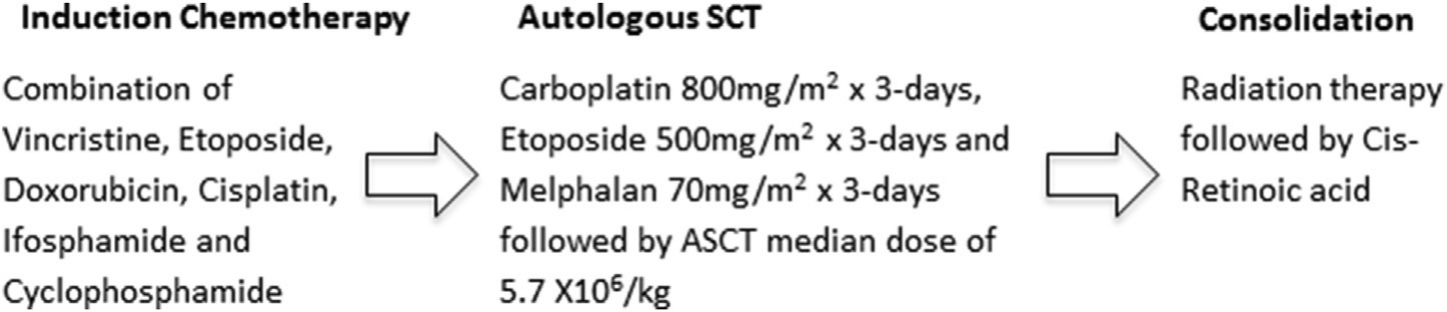
Schematic of treatment plan.

All patients were staged by computed tomography (CT) scan, bone scan, bone marrow, and N-MYC examination when possible. Patients were assigned a risk group according to the Children Oncology Group stratification. Average therapy was completed within a year and a half. Induction therapy for these children included five courses of chemotherapy, including combinations of vincristine, doxorubicin, etoposide, cisplatin, ifosphamide, and cyclophosphamide as per our institutional protocol (Fig. 1).

Follow-up evaluation, including CT scan, bone scintigraphy, bone marrow, and metaiodobenzylguanidine scan when available, was then repeated at the end of the fifth cycle of chemotherapy to evaluate treatment response before surgical resection of the tumor.

Treatment response was determined by the treating physicians and radiologists, who evaluated imaging studies during the institutional multidisciplinary team meetings. In our institution, complete response (CR) was deemed when no evidence of primary tumor and metastatic lesions were seen. Very good partial response (VGPR) was considered when the primary tumor’s size decreased by 90%–99% with no metastatic lesions (except bone), no new bone lesions along with improvement in preexisting lesions. A partial response (PR) was characterized by a decrease in the primary tumor’s size of 50% with no new metastatic lesions and 50%–90% reduction in preexisting lesion sites. Mixed response (MR) was categorized as no new lesions, more than 50% decrease in primary and less than 50% decrease in other metastatic lesions. Stable disease (SD) was termed when there were no new lesions at primary and metastatic sites with less than 50% reduction and/or less than 25% increase in existing lesions. Lastly, progressive disease (PD) included those that developed any new lesion or those with an increase of measurable lesions by 25%.

Therapy-related adverse effects such as febrile neutropenia, mucositis, diarrhea and hepatorenal toxicity were evaluated according to the Common Terminology Criteria for Adverse Events (CTCAE) guide. Once the treatment protocol was completed, the patients were followed up in outpatient clinics every 3–4 months for examination and routine follow-up laboratory tests.

### 2.1 Data management and statistical considerations

All data were electronically entered into IBM-SPSS (version 20). A nonparametric median test was utilized to test for the significance of differences between two continuous variables after testing for the normality assumptions using Shapiro-Wilk test. Kaplan-Meier survival analysis was carried out to estimate survival outcomes. Breslow (Generalized Wilcoxon) test was utilized to test for the significance of differences between the survival times. Overall survival (OS) was defined as the time between the infusion to the last contact with the healthcare team or death. Event-free survival (EFS) was defined as the time to relapse, PD or death, whichever comes first.

### 2.2 Ethical consideration

The data from the patient’s medical records were collected and maintained in accordance with the institutional policy on data confidentiality, security, and safety. The study was a retrospective review; hence, no consent/assent was taken from patients/parents. A waiver of informed consent/assent was granted by the Institutional Review Board of the hospital.

## 3 Results

A total of 105 patients were transplanted at our institution during the study period. Among these, six patients were excluded from the analysis because they underwent transplant for recurrent NBL after other treatment modalities had failed. We then analyzed 99 patients for our results. The male to female ratio in the cohort was 1:0.7. The median age at diagnosis was 3.0 years (range 0.3–13.6 years) and the same for ASCT was 3.8 years (range, 0.9–14.2 years). Median time to ASCT was 0.6 years (range, 0.4–11.9 years). Clinical characteristics of these HR-NBL patients are listed in Table 1.

**Table 1:** Patient characteristics at initial diagnosis (n = 99).

	n (%)
Age at diagnosis	
<12 months	9 (9.1)
12–18 months	9 (9.1)
≥18 months	81 (81.8)
Gender	
Male	59 (60.6)
Female	40 (40.4)
Primary tumor site	
Adrenal	82 (82.8)
Abdomen/pelvis	13 (13.1)
Others	4 (4.0)
Stage of disease at diagnosis	
Stage IIB	2 (2.0)
Stage III	8 (8.1)
Stage IV	87 (87.9)
Stage IVS	2 (2.0)
N-MYC Status (n = 86)	
Amplified	34 (39.5)
Nonamplified	52 (60.5)
Source of stem cells	
Peripheral blood stem cells	57 (57.6)
Bone marrow	42 (42.4)
Cytoreduction regimen used	
Carboplatin/etoposide/melphalan	64 (64.6)
Carboplatin/etoposide/melphalan/TBI	34 (34.3)
Carboplatin/wtoposide/cyclophosphamide	1 (1.0)

TBI: Total body irradiation.

The most common sites of metastases were the bone and bone marrow. Half of the patients (n = 52) had either undifferentiated or poorly differentiated tumor histology. This was followed by local surgical control. There were 91 patients (92%) who underwent local surgical control. Eight of the patients were not amenable to surgery and hence received radiation therapy (XRT) only. Complete resection was accomplished in 61 patients (67%), partial resection in 28 patients (31%), and surgical information was missing for 2 patients (2%).

Consolidation with ASCT was accomplished using the carboplatin-etoposide-melphalan (CEM) backbone regimen in most patients (n-98 and 99%). Among all patients, 57 (58%) received XRT. Forty patients received radiation to the primary site, 6 patients received radiation to the metastatic sites and 11 were given radiation to both primary and metastatic sites. An XRT dose of 23.4 Gy was typically given to primary and metastatic tumor sites with a boost of 30.6 Gy if needed for macroscopic residual disease.

CR and VGPR were seen in 17 (17%) and PR was seen in 70 (71%) patients before proceeding with ASCT. Five patients had MR, 4 had SD and 3 patients were deemed to have PD. All patients underwent a single ASCT.

The median CD34 dose harvested was 5.7 × 10^6^/kg (1.6–32.8). Total body irradiation (TBI), as part of the conditioning regimen, was administered only to those patients who were treated earlier on in our cohort. Our chemotherapy conditioning regimen did not contain Busulfan, and hence, there was only 1 clinical scenario of venocclusive disease in our patient cohort. There were 3 patients diagnosed with interstitial pneumonitis, 2 with acute encephalopathy, and 3 with hemorrhagic cystitis post-transplant. All patients developed acute episodes of fever and neutropenia. Details of commonly seen toxicities with ASCT consolidation are shown in Table 2.

**Table 2:** Nonhematological toxicity profile for patients receiving single ASCT.

Transplant-related toxicity [1]	n (%)
Mucositis	90 (90.9)
Bacteremia	15 (15.2)
Viral infections	7 (7.1)
Fungal infections	4 (4.0)
Acute renal injury	5 (5.1)
Interstitial pneumonia	3 (3.0)
Seizures	3 (3.0)
Encephalopathy	2 (2.0)
Hemorrhagic cystitis	3 (3.0)
Venocclusive disease	1 (1.0)

Toxicities reported were based on definitions from version 4 of the National Cancer Institute Common Terminology Criteria for Adverse Events [13].

All patients were treated with granulocyte colony-stimulating factor after ASCT for count recovery. Median time to ANC recovery was 11 days and median time to platelet recovery was 30.5 days. Time for ANC recovery with bone marrow as a graft source was prolonged but not statistically significant when compared with PBSCs. Cis-retinoic acid was given to 73 (74%) patients as postconsolidation therapy. Those with PD prior to maintenance chemotherapy were not given cis-retinoic acid.

Four of our patients (4%) among the total cohort were diagnosed with nonhematological secondary malignancies. These included dentinogenic ghost cell tumor, follicular neoplasm of the thyroid, osteochondroma, and ganglioneuroma. Overt secondary leukemia was not detected in our patients while on follow-up with us.

At a median follow-up of 50.2 (11.4) months (95% CI: 27.9–72.4), we lost 26 patients (26.3%). The majority of these patients were more than 18 months of age (n = 24 and 92%). The most common cause that leads to death was disease progression (n = 20 and 76.9%). The remaining of the patients (n = 6, 23.1%) died due to septicemia (n = 4), renal failure leading to multiorgan failure (n = 1), and viral pneumonia later manifesting into acute respiratory distress syndrome (ARDs).

**Fig. 2:**
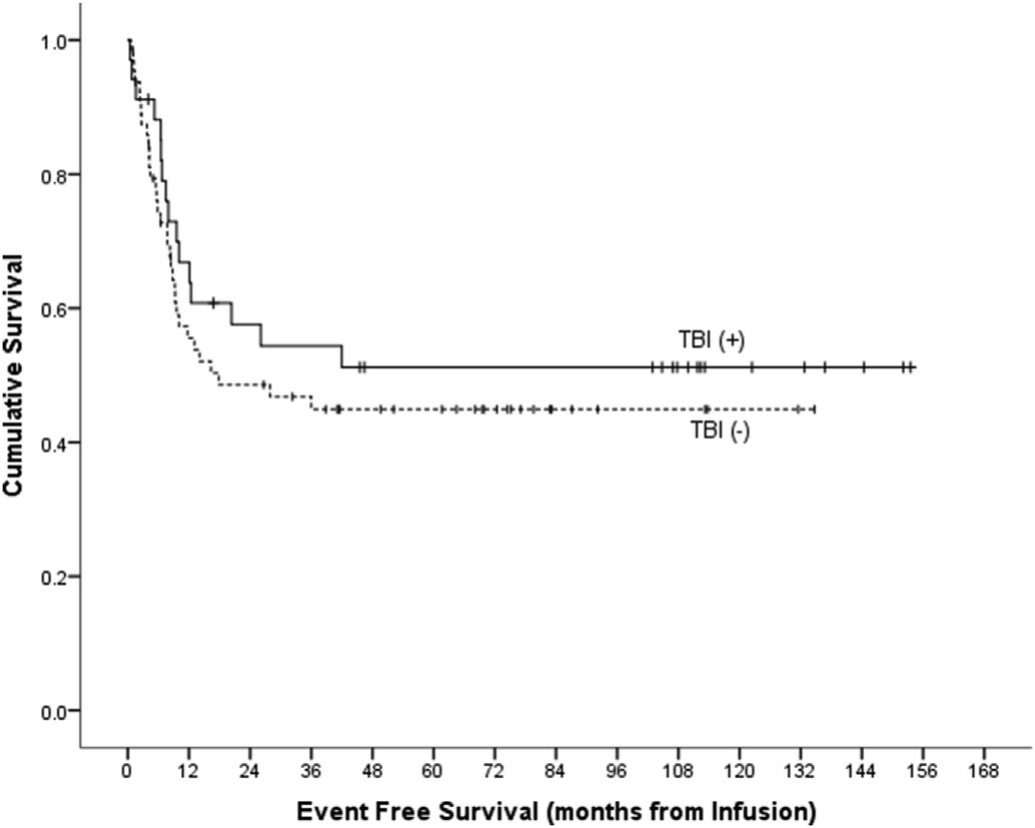
Event-free survival by TBI.

In our analysis, the median time from diagnosis to relapse was 15 months with most patients (n = 37 and 86%) relapsing within two years of diagnosis. Pattern of relapse seen was mostly diffuse disease with bone involvement. Three-year OS was 68.5% (5.2%) and the 3-year EFS was 48.3% (5.2%). Trend for EFS among patients who received TBI was better but not significant when compared with those who did not receive TBI (54.4% [8.7%] vs. 44.9% [6.5%] and *P* Value: 0.337, (Fig. 2).

## 4 Discussion

Currently, most institutions have adopted ASCT. One major institution still excludes ASCT after dose-intense induction chemotherapy by consolidating with radiation therapy, anti GD2-immunotherapy + granulocyte-macrophage colony-stimulating factor (GM-CSF), and isotretinoin [11]. Given their clinical experience, they have discontinued from the use of ASCT, given the possible delay in the initiation of the more potent and targeted immunotherapy. In their view, ASCT shows a trend toward better EFS without leading to a significant difference in OS [12,13]. Thus, the need to study outcomes of single as well as tandem transplants to evaluate its merits in our indigenous patient population.

In this report, we have reviewed our institutional treatment outcomes and toxicity seen in HR-NBL patients treated with induction chemotherapy and surgery followed by a single autologous stem cell transplant during consolidation. Three-year EFS of 48.3% was seen in our patient population. We consider EFS important as it can help identify poor outcomes earlier than OS, given the higher probability of mortality associated with relapse in neuroblastoma [[14], [15], [16]]. The EFS results from our analysis are consistent with trials showing that high-dose chemotherapy and ASCT as being superior to less intensive chemotherapy consolidation [17,18].

The use of ASCT has been shown to be beneficial in neuroblastoma. This is one of the few solid tumors when compared with other high-risk diseases such as Ewing’s sarcoma, osteosarcoma, germ cell tumors, brain tumors, and retinoblastoma to have shown response to dose-escalation of chemotherapy [19]. Autologous stem cells enable us to take advantage of the dose-response relationship of multiple chemotherapeutic agents used in the treatment of neuroblastoma [20]. Our patient population tolerated the high-dose chemotherapy very well with no transplant-related mortality. Hence, we anticipate our HR-NBL patients will tolerate an increase in additional dose intensity with tandem transplants.

Park et al., in their recent paper, showed 3-year EFS from the time of randomization of 48.4% (95% CI, 41.0%–55.7%) in the single transplant group, which is similar to ours (48.3%). Their 3-year EFS of 61.6% (95% CI, 54.3%–68.9^%^) in the tandem transplant group was significantly better than the single transplant group [[21], [22], [23]]. While on the other hand, Ladenstein and his colleagues reported a superior 3-year EFS of 50% in HR-NBL patients treated with single busulfan and melphalan transplant as compared to patients treated with the CEM regimen.

Relapse of NBL tends to usually occur within 2 years of therapy [24]. Our results were similar with majority of our patients (86%) relapsing early on. With the current ongoing developments in the therapy of neuroblastoma, we might possibly see an extension in time to relapse [15,25]. Also, mortality secondary to NBL is mostly early on with less than 5% occurring more than three years from diagnosis [24]. We had a similar result with 25 (96%) patient deaths occurring within 3 years from diagnosis and few deaths seen beyond 5 years.

Secondary malignancies are known to occur in NBL with Rubino et al. showing this to be twice as much as the general population (1.1%) [26]. Our patients had a higher frequency (n = 4 and 4%), and we speculate that this could be secondary to the TBI given during conditioning because three of the four patients (75%) were among that group, but our numbers are few and the follow-up is limited.

This study has its limitations, first is the retrospective nature of the study with patients dating back to 1993 with the use of TBI in the earlier years. Second is that there were different therapeutic protocols used over the study period and some of these differences may have impacted the toxicity profile and outcomes. However, all these protocols were based on varied combinations of the same induction chemotherapeutic agents used today; therefore, it is less likely that variations between protocols would have impacted our findings to greater lengths. This difference in approach can be helpful to our healthcare colleagues in the lower middle-income countries where certain chemotherapeutic agents might not be as readily available, yet varied combinations like ours might be used safely. In centers where peripheral blood harvest is not accessible to all, we see that bone marrow harvests can be an alternate safe option.

## 5 Conclusions

In conclusion, given the improvement seen over the years in conjunction with tandem transplants, we have started to study this approach prospectively in our patient cohort along with anti-disialoganglioside therapy. Autologous transplants with high-dose chemotherapy can lead to prolonged hospital stay and frequent admissions for associated complications that lead to increased medical expenses. Therefore, these aspects also need to be studied for the economic impact of tandem transplants on patient families and the healthcare system that depends on available healthcare resources.

## Author statement

**Saadiya Khan:** Validation; Writing - original draft; Writing - review & editing.

**Khalood AlSayyad:** Conceptualization; Data collection; Investigation; Methodology; Validation; Visualization; Writing - original draft; Writing - review & editing.

**Khawar Siddiqui:** Data processing; Formal analysis; Methodology; Validation; Visualization; Writing - original draft; Writing - review & editing.

**Awatif AlAnazi:** Writing - review & editing.

**Amal AlSeraihy:** Writing - review & editing.

**Ali AlAhmari:** Writing - review & editing.

**Hassan ElSolh:** Writing - review & editing.

**Ibrahim Ghemlas:** Writing - review & editing.

**Hawazen AlSaedi:** Writing - review & editing.

**Abdullah AlJefri:** Writing - review & editing.

**Afshan Ali:** Writing - review & editing.

**Ibrahim AlFawaz:** Writing - review & editing.

**Amani AlKofide:** Writing - review & editing.

**Mouhab Ayas:** Conceptualization; Investigation; Methodology; Project administration; Resources; Supervision; Validation; Writing - original draft; Writing - review & editing.

## Declaration

First and second authors contributed equally to the write-up of this study.

## Funding source

This research did not receive any specific grant from funding agencies in the public, commercial, or not-for-profit sectors.

## Ethical statement

This clinical research study was approved by the Institutional Review Board (IRB) of our hospital, which was to be conducted under the international guidelines for the enrollment of human subjects. The data from patients’ medical records were collected and maintained at the Department of Pediatric Hematology/Oncology, in accordance with institutional policy on data confidentiality, security, and safety. As the study was designed as a retrospective review, no consent/assent was taken from patients/parents. A waiver of informed consent/assent was sought from the IRB and was duly granted.

## Declaration of competing interest

Nothing to declare.

## Figures and Tables

**Fig S1:**
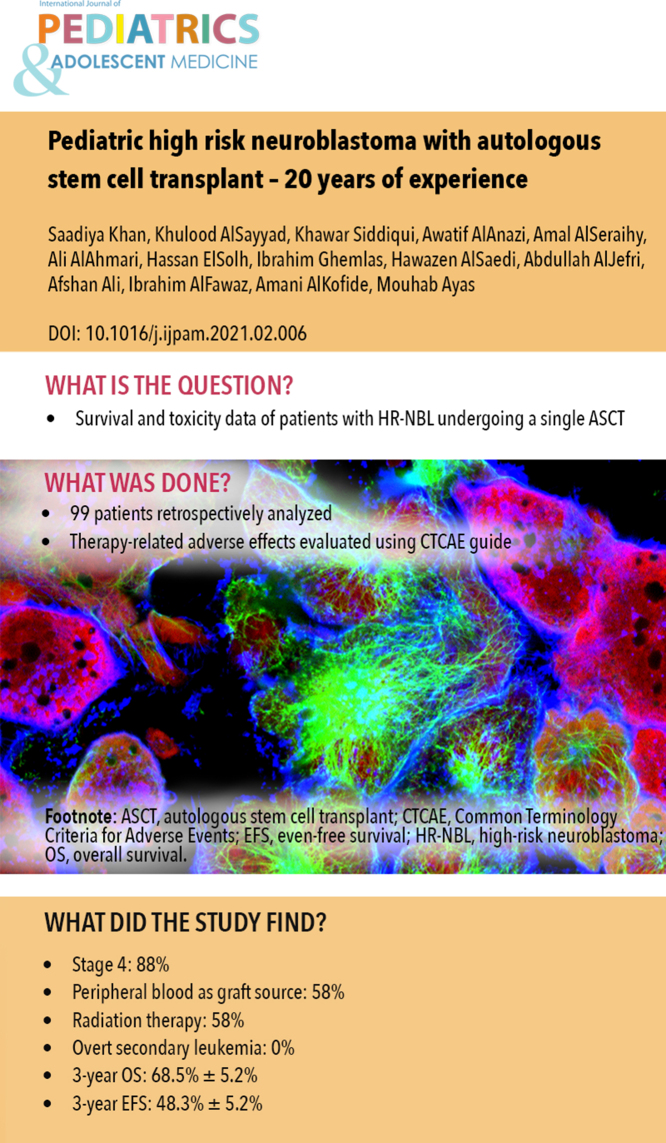


## Visual abstract

The following are the Supplementary data to this article:
